# The Japan Environment and Children’s Study (JECS): A Preliminary Report on Selected Characteristics of Approximately 10 000 Pregnant Women Recruited During the First Year of the Study

**DOI:** 10.2188/jea.JE20140186

**Published:** 2015-06-05

**Authors:** Takehiro Michikawa, Hiroshi Nitta, Shoji F. Nakayama, Masaji Ono, Junzo Yonemoto, Kenji Tamura, Eiko Suda, Hiroyasu Ito, Ayano Takeuchi, Toshihiro Kawamoto

**Affiliations:** 1 National Center for the Japan Environment and Children’s Study, National Institute for Environmental Studies, Tsukuba, Ibaraki, Japan; 1 独立行政法人国立環境研究所 環境健康研究センター エコチル調査コアセンター; 2 Department of Environmental Health, University of Occupational and Environmental Health, Kitakyushu, Fukuoka, Japan; 2 産業医科大学 医学部 産業衛生学

**Keywords:** birth cohort, pregnant women, children, environmental chemicals, Japan

## Abstract

**Background:**

The Japan Environment and Children’s Study (JECS) is an ongoing nationwide birth cohort study launched in January 2011. In this progress report, we present data collected in the first year to summarize selected maternal and infant characteristics.

**Methods:**

In the 15 Regional Centers located throughout Japan, the expectant mothers were recruited in early pregnancy at obstetric facilities and/or at local government offices issuing pregnancy journals. Self-administered questionnaires were distributed to the women during their first trimester and then again during the second or third trimester to obtain information on demographic factors, physical and mental health, lifestyle, occupation, environmental exposure, dwelling conditions, and socioeconomic status. Information was obtained from medical records in the first trimester and after delivery on medical history, including gravidity and related complications, parity, maternal anthropometry, and infant physical examinations.

**Results:**

We collected data on a total of 9819 expectant mothers (mean age = 31.0 years) who gave birth during 2011. There were 9635 live births. The selected infant characteristics (singleton births, gestational age at birth, sex, birth weight) in the JECS population were similar to those in national survey data on the Japanese general population.

**Conclusions:**

Our final birth data will eventually be used to evaluate the national representativeness of the JECS population. We hope the JECS will provide valuable information on the impact of the environment in which our children live on their health and development.

## Abstract (ja)

**【背景】:**

子どもの健康と環境に関する全国調査（エコチル調査）は、2011年に妊婦の参加登録を開始した環境省が主導する全国規模の出生コホート研究である。この調査の進捗を報告するため、我々は開始1年目に集まったデータを用いて、妊婦および出生児の基本属性を集計した。

**【方法】:**

日本全国15地域において、産科施設あるいは自治体の母子健康手帳発行窓口にて参加者（妊婦）をリクルートした。妊娠前期に1回目の質問票調査、妊娠中期から後期に2回目の質問票調査を行い、参加者の基礎情報、身体的精神的な健康状態、生活習慣、職業、生活環境曝露、社会経済状況などの情報を収集した。また、妊娠出産歴、妊娠に関連した病歴、身体計測値、出産記録などの医療情報については、妊娠前期および出産時に実施した診療録転記調査から得た。

**【結果】:**

開始1年目の2011年に出産した妊婦9.819名（平均年齢31.0歳）の基本属性をまとめた。生産児は9,635名であり、その胎数、在胎期間、性比、出生時体重に関する集計結果は、国の統計と非常に近い結果であった。

**【結論】:**

全ての出産を含んだ出産時データがまとまった時、エコチル調査参加者の国民代表性について最終的な検討を行う予定である。このエコチル調査から得られる成果は、日本の環境が今を生きる子ども達の健康や成長に与える影響について有益な情報を与えるものと考えている。

## INTRODUCTION

The Japan Environment and Children’s Study (JECS) is an ongoing nationwide birth cohort study.^1^ Its primary objective is to investigate environmental factors, such as exposure to chemicals and airborne pollutants, that could affect children’s health and development during the fetal stage and early childhood, in order to help policymakers formulate measures to safeguard the environment for future generations.

The plan was to recruit approximately 100 000 pregnant women and their partners over a period of three years, to collect biological samples (blood, urine, hair, breast milk, and umbilical cord blood), and to collect data on their children until they reach 13 years of age. In January 2011, we started recruiting women in study areas nationwide, from Hokkaido in the north to Okinawa in the south. The study areas were selected with the intention of making the results of JECS generalizable. So, to check whether recruitment was going according to plan, we decided to summarize the data we collected so far on selected maternal and infant characteristics. To this end, we used data on approximately 10 000 women who gave birth in 2011 (the first year of recruitment).

## MATERIALS AND METHODS

### Study participants

The JECS protocol has been published elsewhere.^1^ Fifteen Regional Centers (Hokkaido, Miyagi, Fukushima, Chiba, Kanagawa, Koshin, Toyama, Aichi, Kyoto, Osaka, Hyogo, Tottori, Kochi, Fukuoka, and south Kyushu/Okinawa) were selected for their wide geographical distribution, and each was made responsible for recruiting pregnant women who lived in administratively defined districts (city, town, or village) within the relevant study area (eTable 1). The women were recruited in early pregnancy at obstetric facilities and/or at local government offices issuing pregnancy journals (Mother-Child Health Handbooks). Recruitment started in January 2011 and continued for three years, until March 2014.

The present study is based on the data set of jecs-ag-ai-20131008, which was released in October 2013. The study population was 9838 women who gave birth on or before December 31, 2011. Of these, we excluded participants with missing data on maternal age and gestational age at delivery, leaving us with a total of 9819 mothers, including 94 mothers with multiple registrations. The target recruitment rate is >50% of qualified women in the Residential Registry of each study area, and the final rate will be reported when all birth data are confirmed.

### Questionnaires and medical record transcription

The research coordinators distributed self-administered questionnaires at prenatal examinations or by mail to the women during their first trimester (first questionnaire) and again during their second/third trimester (second questionnaire). The completed questionnaires were then returned either by hand at subsequent prenatal visits or by mail. Incomplete answers were supplemented as much as possible through face-to-face or telephone interviews. Table 1 shows the questions used in the two questionnaires to obtain information on demographic factors, medical and obstetric history, physical and mental health, lifestyle, occupation (based on the Japan Standard Occupational Classification, 2009^2^), environmental exposure, dwelling conditions, and socioeconomic status. These questionnaires included the SF-8 as an indicator of health-related quality of life,^3^ the K6 as an indicator of psychological distress,^4^^–^^7^ the short version of the International Physical Activity Questionnaire as an indicator of physical activity,^8^^,^^9^ the Autism Spectrum Quotient-10 as an indicator of autistic traits,^10^^,^^11^ and the food frequency questionnaire used in the Japan Public Health Center-based prospective Study for the Next Generation (JPHC-NEXT). Information was obtained from maternal and infant medical records on medical history, including gravidity and related complications, parity, maternal anthropometry, and infant physical examinations (Table 1). Transcripts of medical records were made twice by physicians, midwives/nurses, or research coordinators, once during each woman’s first trimester and again after delivery. Data collected via the questionnaires and medical transcripts were scanned, converted to electronic data with an optical character recognition device, and then stored in a data management system.

**Table 1.** Summary of information collected by questionnaires and medical record transcription in the Japan Environment and Children’s Study (JECS)

Example of individual item		
Maternal questionnaire at the first trimester (the first questionnaire)		
	Demographic factors	date of birth, age, marital status, family structure
	Medical and obstetric history	
	Physical and mental health	height and weight before pregnancy, health-related quality of life as measured with the SF-8,^3^ psychological distress using the K6^4^^–^^7^
	Lifestyle	smoking habits (including partner’s status), passive smoking, alcohol consumption, dietary habits before pregnancy using the Food Frequency Questionnaire, physical activity using the short version of the International Physical Activity Questionnaire^8^^,^^9^
	Occupation	
	Environmental exposure at home and in workplace	

Maternal questionnaire in the second/third trimester (the second questionnaire)		
	Demographic factors	date of birth, age
	Physical and mental health	health-related quality of life as measured with the SF-8,^3^ psychological distress using the K6,^4^^–^^7^ autistic traits as measured with the Autism Spectrum Quotient-10,^10^^,^^11^ life events
	Lifestyle	smoking habits (including partner’s status), passive smoking, alcohol consumption, dietary habits during pregnancy using the Food Frequency Questionnaire, dietary supplement intake during pregnancy, physical activity using the short version of the International Physical Activity Questionnaire,^8^^,^^9^ sleeping
	Dwelling conditions	condition of one’s home, use of air conditioning system, presence of visible mold, pets, frequency of vacuuming, use of chemical substances such as insecticide and repellent
	Occupation	
	Environmental exposure at home and in workplace	
	Socioeconomic status	educational background (including partner’s status), household income, social capital

Medical record transcription at the first trimester		
	Demographic factors	date of birth, age
	Anthropometry	height and weight
	Medical history of present pregnancy	expected date of confinement, infertility treatment, fetal number
	Medical history of past pregnancies	

Medical record transcription at birth		
	Maternal	
	Demographic factors	date of birth, age
	Medical history	medical and pregnancy complications, infection such as hepatitis virus, medication
	Infant	
	Birth record	date of birth, gestational age, number of live births, type of delivery, fetal presentation, sex, apgar score, anthropometry, neonatal complications
	Physical examinations	congenital anomalies

### Ethical issues

The JECS protocol was reviewed and approved by the Ministry of the Environment’s Institutional Review Board on Epidemiological Studies and by the Ethics Committees of all participating institutions. Written informed consent was obtained from all participating women.

### Statistical analysis

For the present study, we obtained information about maternal age at delivery, marital status, educational background, household income, smoking habits, passive smoking, alcohol consumption, and history of obstetrical/gynecological diseases from the questionnaires; height and weight before pregnancy, parity, and infertility treatment from both questionnaire and medical record; delivery outcomes (live birth or not, singleton or multiple birth, gestational age at birth, sex, type of delivery, and birth weight) from medical records.

Since both the first and second questionnaires included questions about smoking and alcohol consumption, we used data from the first questionnaire, with supplementary data added from the second questionnaire. We divided smoking status into three categories: never smoked, ex-smokers who quit before pregnancy, smokers during early pregnancy (ie, those who responded “quit after pregnancy was confirmed” or “still smoking”). Alcohol consumption status was divided into three categories in the first questionnaire (never, used to drink, or still drinking), and into four categories in the second questionnaire (never, quit drinking before pregnancy was confirmed, quit drinking after pregnancy was confirmed, or still drinking). From the two sets of responses, we made three groups: never drank, ex-drinkers who quit before pregnancy (those whose cessation after pregnancy was confirmed were not classed as ex-drinkers), or drinkers during early pregnancy. The medical record data on maternal height and weight before pregnancy, parity, and infertility treatment were used when available, with the information collected via questionnaire used only as a fallback measure. Body mass index (BMI) before pregnancy was calculated as weight (kg)/height squared (m^2^).

The selected characteristics were summarized according to maternal age group (<25, 25–29, 30–34, or ≥35 years) and Regional Center. All analyses were performed with Stata 11 (StataCorp LP, College Station, TX, USA).

## RESULTS

Of the 9819 women, 10.7% were 24 years old or younger, 28.1% were 25 to 29 years old, 35.2% were 30 to 34 years old, and 26.0% were aged 35 years or older; the mean age (standard deviation [SD]) was 31.0 (5.0) years. The first questionnaire was returned by 9563 of the women (97.4%), and the second questionnaire was returned by 9369 (95.4%). As shown in Table 2, the majority of the women were married (95.8%), well-educated (63.2% with ≥13 years of education), and had an appropriate BMI before pregnancy of 18.5 to 24.9 kg/m^2^ (72.8%). Smokers during early pregnancy accounted for 19.3% of the participants, and 43.8% drank alcohol during early pregnancy. The most common pregnancy-related abnormality was pregnancy-induced hypertension (3.6% among parous women). The proportions of nulliparae and primiparae did not differ substantially (40.9% vs 38.2%). Missing information on household income was more common (10.6%) than that for other variables.

**Table 2.** Selected maternal characteristics in the Japan Environment and Children’s Study (JECS) as of 2011

Variables	*n*^a^		Age at delivery, years			

		Total	<25	25–29	30–34	≥35

		(%)	(%)	(%)	(%)	(%)
Number of mothers	9819	100	1049	2762	3456	2552
Age at delivery, years						
Total, mean (SD)	9819	31.0 (5.0)				
<25		10.7				
25–29		28.1				
30–34		35.2				
≥35		26.0				
Marital status	9521					
Married		95.8	84.5	95.2	98.1	97.7
Unmarried		3.3	14.3	3.7	1.3	1.1
Divorced/widowed		1.0	1.2	1.2	0.6	1.1
Educational background, years	9326					
<10		5.2	20.4	5.2	2.5	2.6
10–12		31.7	54.0	34.8	25.2	28.2
13–16		61.8	25.4	59.2	70.5	67.4
≥17		1.4	0.2	0.9	1.9	1.7
Household income, million Japanese Yen	8780					
<2		6.1	18.2	7.1	3.7	3.9
2 to <4		35.2	51.8	42.7	32.4	24.7
4 to <6		33.0	20.7	30.8	36.2	35.5
6 to <8		15.1	4.9	12.1	16.6	20.2
8 to <10		6.4	1.8	4.3	7.3	9.0
≥10		4.2	2.6	2.9	3.9	6.7
Smoking habits	9599					
Never smoked		56.5	47.4	54.3	58.9	59.2
Ex-smokers who quit before pregnancy		24.2	17.8	22.8	25.7	26.4
Smokers during early pregnancy		19.3	34.8	22.9	15.5	14.4
Passive smoking	9520					
Rarely		47.3	24.3	41.3	53.4	54.8
Daily		19.9	37.3	22.0	16.2	15.9
Alcohol consumption	9609					
Never drank		35.2	36.1	36.8	35.7	32.2
Ex-drinkers who quit before pregnancy		21.0	21.1	19.9	21.2	22.0
Drinkers during early pregnancy		43.8	42.8	43.3	43.1	45.8
Body mass index before pregnancy, kg/m^2^	9768					
<18.5		16.7	20.3	19.0	16.9	12.2
18.5–24.9		72.8	70.5	71.9	71.9	75.9
≥25.0		10.6	9.2	9.2	11.2	11.8
History of obstetrical/gynecological diseases	9563					
Dysmenorrhea		11.0	10.7	11.5	11.9	9.6
Endometriosis		3.6	1.6	2.6	3.8	5.3
Myoma uteri		6.2	0.4	2.2	6.2	12.6
Adenomyosis uteri		0.2	0.0	0.2	0.2	0.4
Malformation of uterus		0.3	0.1	0.3	0.4	0.3
Ovarian tumor/cyst		3.4	1.7	2.7	3.6	4.7
Polycystic ovary syndrome		1.6	1.1	1.6	1.6	1.6
Urogenital malformation		0.03	0.0	0.0	0.09	0.0
Pregnancy-induced hypertension^b^		3.6	3.8	2.5	3.7	4.2
Gestational diabetes mellitus^b^		0.8	0.6	0.5	0.8	1.2
Placenta abruption^b^		0.5	0.0	0.2	0.6	0.6
Ectopic pregnancy^b^		0.9	1.1	0.6	0.9	1.2
Placenta previa^b^		0.7	0.0	0.4	0.5	1.4
Hydatidiform mole^b^		0.6	0.6	0.7	0.8	0.5
Parity	9658					
0		40.9	62.4	49.7	35.7	29.8
1		38.2	30.3	36.5	41.9	38.4
≥2		20.8	7.4	13.8	22.4	31.9
Infertility treatment	9792					
No		94.4	99.7	97.1	94.6	89.3

SD, standard deviation.

^a^Number of mothers without missing value.

^b^Parous women only (*n* = 5581).

The selected maternal characteristics according to age groups are shown in Table 2. The younger age groups tended to have higher proportions of smokers during early pregnancy, daily passive smokers, underweight before pregnancy (BMI <18.5 kg/m^2^), nulliparae, and participants without infertility treatment. There were no substantial differences among age groups in regard to alcohol consumption. The maternal characteristics according to Regional Center are summarized in eTable 2.

From 9819 deliveries in 2011, a total of 9635 live births and 26 stillbirths (fetal deaths that occurred at ≥22 weeks of gestation) were observed. Of 9635 live births, the proportion of singleton births was 98.2%, the mean gestational age (SD) at birth was 39.0 (1.8) weeks, the proportion of term births was 92.9%, and 51.0% were male (Table 3). Approximately 80% of the births were by vaginal delivery, with the proportion declining in proportion to age. With regard to singleton births, the mean birth weight (SD) was 3002 (433) g. The distribution of low birth weight (birth weight <2500 g) did not differ substantially across the age groups. The distributions of infant characteristics according to Regional Center are shown in eTable 3.

**Table 3.** Selected infant characteristics in the Japan Environment and Children’s Study (JECS) as of 2011

Variables	*n*^a^		Maternal age at delivery, years			

		Total	<25	25–29	30–34	≥35

		(%)	(%)	(%)	(%)	(%)
Number of live births	9635	100	1021	2730	3404	2480
Singleton births		98.2	98.6	98.0	98.1	98.3
Gestational age at birth, weeks						
Total, mean (SD)	9635	39.0 (1.8)	39.2 (1.7)	39.1 (1.8)	39.1 (1.7)	38.9 (1.9)
Preterm births (<37)		6.9	6.5	7.4	5.6	8.2
Term births (37–41)		92.9	93.4	92.4	94.1	91.7
Postterm births (≥42)		0.2	0.1	0.2	0.3	0.2
Sex	9635					
Male		51.0	52.3	51.7	50.1	51.1
Female		48.8	47.2	48.0	49.7	48.8
Missing^b^		0.2	0.5	0.3	0.2	0.1
Type of delivery	9604					
Vaginal		80.3	86.8	84.0	79.8	74.2
Cesarean		19.7	13.2	16.0	20.2	25.8
Birth weight, g						
Total, mean (SD)	9607	2987 (447)	2981 (425)	2979 (441)	2997 (441)	2985 (471)
Singleton births	9430					
Total, mean (SD)		3002 (433)	2992 (413)	2997 (424)	3012 (427)	2997 (459)
Male, mean (SD)		3046 (431)	3050 (421)	3041 (410)	3052 (437)	3044 (449)
Female, mean (SD)		2956 (430)	2929 (396)	2952 (433)	2973 (412)	2948 (464)
Low birth weight (<2500 g)		9.1	9.8	8.5	9.1	9.4

SD, standard deviation.

^a^Number of infants without missing value.

^b^Including newborns with ambiguous genitalia.

## DISCUSSION

This is a preliminary report on selected maternal and infant characteristics of the approximately 10 000 pregnant women who gave birth during the first year of recruitment (2011) for the JECS.

The JECS study areas encompass the whole of Japan from north to south. We wanted to ensure the study population was representative of the general population, so we compared selected characteristics of the JECS population with those obtained via a national survey (Table 4).^12^^–^^14^ In the Vital Statistics survey,^12^ the proportion of nulliparae was determined on the basis of birth order data, so the comparison of Vital Statistics with our results of parity should be done with caution. However, we think the proportion of nulliparae was probably lower in the JECS than in the general population because caution on the part of women in their first pregnancy may have made them hesitate to participate in the present study. There were no essential differences in the distribution of maternal age at delivery between the JECS participants and the general population. The proportions of JECS women aged 20–29 years and those aged 30–39 years were 37.8% and 57.1%, respectively; the corresponding figures for Vital Statistics 2011 were 38.5% and 56.6%.^12^ Fetal death rate at ≥22 weeks’ gestation of the JECS population (2.7 per 1000 live births and fetal deaths at ≥22 weeks) did not substantially differ from that of the general population (3.3 per 1000 live births and fetal deaths at ≥22 weeks for Vital Statistics 2011^12^). The proportions of singleton births, term births, cesarean births, and low birth weight were also similar between the two sets of data.^13^^,^^14^ In addition, the infant male:female ratios were comparable (1.04 for the JECS and 1.05 for Vital Statistics 2011^12^). As of 2011 (the first year of recruitment), the JECS cohort can be regarded as representative of the Japanese general population. The final birth data will eventually be used to evaluate the national representativeness of the JECS population.

**Table 4.** Selected maternal and infant characteristics of the Japan Environment and Children’s Study (JECS) and Japanese national surveys

	JECS	National survey	
	
	(%)	(%)	
Maternal characteristics			
Age at delivery, years			Vital Statistics, 2011^12^
20–29	37.8	38.5	
30–39	57.1	56.6	
Parity			
0	40.9	^a^	
Infant characteristics			
Live births			Birth Statistics, 2010^13^
Singleton births	98.2	98.0	
Gestational age at birth, weeks			Birth Statistics, 2010^13^
Term births (37–41)^b^	93.9	94.9	
Sex^c^			Vital Statistics, 2011^12^
Male	51.1	51.2	
Female	48.9	48.8	
Type of delivery			Surveys of Medical Institutions, 2011^14^
Cesarean	19.7	19.2	
Birth weight^b^			Birth Statistics, 2010^13^
Total, mean kg	3.00	3.02	
Low birth weight (<2500 g)	9.1	8.3	

^a^In Vital Statistics,^12^ birth order has been reported. The proportion of first child among the number of the total births was 47.1% in 2011.

^b^Singleton births only.

^c^Excluding missing data.

Smoking and alcohol consumption are major lifestyle factors strongly suspected of contributing to adverse birth outcomes.^15^ We are separately researching the effects of such maternal lifestyle factors on children’s health and development, although JECS puts primary emphasis on involuntary exposure to environmental factors. We compared the lifestyle characteristics of our study population with corresponding data on the general population, but there is actually little nationwide information about smoking and alcohol consumption among Japanese pregnant women. In a 2006 survey at the medical institutions specified by the Japan Association of Obstetricians and Gynecologists for a survey of infectious disease and statistics throughout Japan,^16^^,^^17^ approximately 19 000 expectant mothers at various stages of pregnancy were asked about their smoking and drinking habits before and after pregnancy. It is difficult to compare the results of this survey with our data with respect to smoking/drinking status during early pregnancy, but the distributions of smokers and drinkers by age groups tended to be similar in the two studies. A prospective cohort study of pregnant women and their children carried out in Koshu City, Project Koshu,^18^ demonstrated similar results: the proportion of smokers during early pregnancy was 21.5% (current smokers [6.6%] + those who quit during early pregnancy [14.9%]); our finding was 19.3%.

JECS recruitment of participants finished at the end of March 2014 after the registration of approximately 100 000 women. After the last participants have given birth in the end of 2014, we will be able to establish the largest birth cohort ever assessed in Japan, which will also be one of the largest globally. The results regarding factors associated with the pregnancy and birth outcomes are forthcoming. The participating children will be followed until they reach 13 years of age, and we expect that the JECS will provide valuable information on the impact of the environments in which our children live on their health and development.

## ONLINE ONLY MATERIALS

eTable 1. Study areas of each Regional Center in the Japan Environment and Children’s Study (JECS) as of the start of recruitment (January 2011).

eTable 2. Selected maternal characteristics according to Regional Centers in the Japan Environment and Children’s Study (JECS) as of 2011.

eTable 3. Selected infant characteristics according to Regional Centers in the Japan Environment and Children’s Study (JECS) as of 2011.

Abstract in Japanese.

## APPENDIX

Members of the Japan Environment and Children’s Study (JECS) as of 2014 (principal investigator, Toshihiro Kawamoto): Hirohisa Saito (Medical Support Center for JECS, National Center for Child Health and Development, Tokyo, Japan), Reiko Kishi (Hokkaido Regional Center for JECS, Hokkaido University, Sapporo, Japan), Nobuo Yaegashi (Miyagi Regional Center for JECS, Tohoku University, Sendai, Japan), Koichi Hashimoto, Seiji Yasumura (Fukushima Regional Center for JECS, Fukushima Medical University, Fukushima, Japan), Chisato Mori (Chiba Regional Center for JECS, Chiba University, Chiba, Japan), Fumiki Hirahara (Kanagawa Regional Center for JECS, Yokohama City University, Yokohama, Japan), Zentaro Yamagata (Koshin Regional Center for JECS, University of Yamanashi, Chuo, Japan), Hidekuni Inadera (Toyama Regional Center for JECS, University of Toyama, Toyama, Japan), Michihiro Kamijima (Aichi Regional Center for JECS, Nagoya City University, Nagoya, Japan), Ikuo Konishi (Kyoto Regional Center for JECS, Kyoto University, Kyoto, Japan), Hiroyasu Iso (Osaka Regional Center for JECS, Osaka University, Suita, Japan), Masayuki Shima (Hyogo Regional Center for JECS, Hyogo College of Medicine, Nishinomiya, Japan), Munetsugu Fukumoto (Tottori Regional Center for JECS, Tottori University, Yonago, Japan), Narufumi Suganuma (Kochi Regional Center for JECS, Kochi University, Nankoku, Japan), Toshiro Hara (Fukuoka Regional Center for JECS, Kyushu University, Fukuoka, Japan), and Takahiko Katoh (South Kyushu/Okinawa Regional Center for JECS, Kumamoto University, Kumamoto, Japan).
