# Supplementary material for: The Japan Environment and Children’s Study (JECS): A Preliminary Report on Selected Characteristics of Approximately 10 000 Pregnant Women Recruited During the First Year of the Study
Source: J Epidemiol. 2015 Jun 5;25(6):452–8. doi: 10.2188/jea.JE20140186 (PMC4444500; doi:10.2188/jea.JE20140186)
Supplement: eTable 1. [file je-25-452-s001.pdf]

eTable 1. Study areas of each Regional Center in the Japan Environment and Children's Study (JECS) as of the start of recruitment (January 2011)

Regional Center	Study area
Hokkaido	Kita-ku and Toyohira-ku in Sapporo City, part of Asahikawa City, part of Kitami City, Oketo Town, Kunneppu Town, Tsubetsu Town, and Bihoro Town
Miyagi	Kesennuma City, Minamisanriku Town, Ishinomaki City, Onagawa Town, Osaki City, Wakuya Town, Misato Town, Kami Town, Shikama Town, Kurihara City, Tome City, Iwanuma City, Watari Town, and Yamamoto Town
Fukushima	Fukushima City, Minamisoma City, Namie Town, Futaba Town, Okuma Town, Katsurao Village, Tomioka Town, Naraha Town, Hirono Town, and Kawauchi Village
Chiba	Kamogawa City, Minamiboso City, Tateyama City, Kyonan Town, Katsuura City, Isumi City, Onjuku Town, Otaki Town, Kisarazu City, Sodegaura City, Futtsu City, Kimitsu City, and Midori-ku in Chiba City
Kanagawa	Kanazawa-ku Yokohama City, Yamato City, and Odawara City
Koshin	Kofu City, Chuo City, Koshu City, Yamanashi City, Fujiyoshida City, Ina City, Komagane City, Tatsuno Town, Minowa Town, Iijima Town, Minamiminowa Village, Nakagawa Village, and Miyada Village
Toyama	Toyama City, Kurobe City, Asahi Town, and Nyuzen Town
Aichi	Ichinomiya City and Kita-ku in Nagoya City
Kyoto	Sakyou-ku and Kita-ku in Kyoto City, Kizugawa City, and Nagahama City
Osaka	Kishiwada City, Kaizuka City, Kumatori Town, Izumisano City, Tajiri Town, Sennan City, Hannan City, and Misaki Town
Hyogo	Amagasaki City
Tottori	Yonago City, Sakaiminato City, Daisen Town, Houki Town, Nanbu Town, Kofu Town, Hino Town, Nichinan Town, and Hiezu Village
Kochi	Kochi City, Nankoku City, Shimanto City, and Yusuhara Town
Fukuoka	Yahatanishi-ku in Kitakyushu City, and Higashi-ku in Fukuoka City
South Kyushu/Okinawa	Minamata City, Tsunagi Town, Ashikita Town, Amakusa City, Reihoku Town, Kamiamakusa City, Hitoyoshi City, Nishiki Town, Asagiri Town, Taragi Town, Yunomae Town, Mizukami Village, Sagara Village, Itsuki Village, Yamae Village, Kuma Village, Nobeoka City, and Miyakojima City
