# Supplementary material for: The Japan Environment and Children’s Study (JECS): A Preliminary Report on Selected Characteristics of Approximately 10 000 Pregnant Women Recruited During the First Year of the Study
Source: J Epidemiol. 2015 Jun 5;25(6):452–8. doi: 10.2188/jea.JE20140186 (PMC4444500; doi:10.2188/jea.JE20140186)
Supplement: eTable 2. [file je-25-452-s002.pdf]

eTable 2. Selected maternal characteristics according to Regional Centers in the Japan Environment and Children's Study (JECS) as of 2011

Variables[a]	Hokkaido (%)	Miyagi (%)	Fukushima (%)	Chiba (%)	Kanagawa (%)	Koshin (%)	Toyama (%)	Aichi (%)	Kyoto (%)	Osaka (%)	Hyogo (%)	Tottori (%)	Kochi (%)	Fukuoka (%)	South Kyushu /Okinawa (%)
Number of mothers	659	1,067	589	546	483	848	714	471	165	1,067	505	315	781	889	720
Age at delivery, years															
Total, mean (SD)	31.2 (4.7)	29.8 (5.3)	30.5 (4.9)	30.6 (5.2)	31.5 (5.1)	31.3 (4.8)	31.6 (4.8)	32.0 (4.8)	32.3 (4.4)	30.6 (5.2)	31.6 (4.9)	31.0 (4.9)	31.6 (4.9)	31.0 (5.0)	30.3 (5.0)
<25	8.4	16.9	13.2	12.5	8.7	8.1	6.9	6.8	6.1	13.2	8.3	10.8	6.9	10.8	13.8
25-29	27.3	32.0	30.1	28.8	26.9	27.0	27.5	21.7	18.8	27.7	27.3	27.3	27.8	29.4	30.8
30-34	39.2	31.4	34.1	33.9	34.8	38.0	35.3	39.1	43.0	34.3	36.0	35.6	35.3	34.4	33.1
≥35	25.2	19.8	22.6	24.9	29.6	26.9	30.4	32.5	32.1	24.8	28.3	26.4	30.0	25.4	22.4
Marital status															
Married	96.5	94.0	95.7	96.3	97.4	96.5	97.4	97.8	98.6	94.0	97.1	95.1	95.0	94.3	96.3
Unmarried	2.9	4.5	3.4	3.1	2.4	2.9	2.3	1.5	1.4	4.3	2.1	2.9	3.9	4.2	2.7
Divorced/widowed	0.6	1.4	0.9	0.6	0.2	0.6	0.3	0.7	0.0	1.7	0.8	2.0	1.1	1.5	1.0
Educational background, years															
<10	3.5	6.2	4.3	7.1	3.7	4.7	2.7	3.5	0.7	8.0	5.2	5.3	4.3	6.3	6.1
10-12	31.0	48.2	36.1	33.8	24.1	26.0	22.7	26.4	16.3	31.5	26.2	32.0	24.9	28.2	45.6
13-16	63.9	45.0	59.1	57.5	68.8	68.2	72.4	68.1	78.2	59.9	66.9	62.0	69.2	63.6	48.0
≥17	1.6	0.6	0.5	1.7	3.4	1.1	2.2	2.0	4.8	0.6	1.7	0.7	1.6	1.9	0.3
Household income, million Japanese Yen															
<2	5.4	10.1	4.2	5.1	2.3	4.0	2.0	1.8	0.7	7.1	3.9	5.9	9.4	6.5	12.8
2 to <4	35.6	39.3	38.3	32.6	25.7	38.1	25.3	24.8	32.4	37.4	28.9	38.8	34.7	35.5	48.2
4 to <6	35.1	29.5	32.1	32.6	37.4	32.9	37.4	37.5	34.5	34.1	36.0	29.9	30.4	34.3	25.7
6 to <8	15.0	10.8	14.6	15.9	20.5	13.7	22.2	20.1	16.6	13.5	19.1	18.1	15.8	14.2	7.7
8 to <10	5.7	5.7	6.0	7.1	8.8	6.5	8.5	10.2	7.2	5.6	7.7	3.7	7.1	5.0	2.9
≥10	3.3	4.7	4.7	6.9	5.4	4.7	4.5	5.6	8.6	2.3	4.3	3.7	2.6	4.5	2.8
Smoking habits															
Never smoked	45.9	49.8	54.9	49.0	61.4	57.3	59.0	69.0	71.1	56.9	60.3	60.0	59.9	56.4	56.4
Ex-smokers who quit before pregnancy	30.8	23.4	25.3	28.3	24.8	25.1	25.8	21.7	22.2	21.8	25.3	25.5	21.1	22.6	22.2
Smokers during early pregnancy	23.3	26.8	19.7	22.7	13.8	17.7	15.2	9.3	6.7	21.2	14.5	14.5	19.0	21.0	21.4
Passive smoking															
Rarely	47.4	34.8	44.3	42.2	51.3	54.0	53.5	52.7	73.9	43.2	49.0	52.3	51.5	47.3	44.7
Daily	19.6	32.0	21.9	22.7	13.3	16.6	15.9	10.9	9.2	23.5	14.9	15.6	17.7	20.6	21.0
Alcohol consumption															
Never drank	26.3	35.6	33.0	37.3	34.2	35.6	38.1	40.4	40.0	39.5	34.8	39.4	32.3	35.2	30.9
Ex-drinkers who quit before pregnancy	18.2	23.2	15.8	27.5	15.0	23.0	17.7	22.8	27.3	19.2	23.4	28.1	23.0	18.8	21.3
Drinkers during early pregnancy	55.6	41.2	51.2	35.2	50.8	41.4	44.3	36.8	32.7	41.3	41.8	32.6	44.7	46.1	47.7
Body mass index before pregnancy, kg/m^2															
<18.5	18.1	14.2	14.6	14.0	16.4	16.6	18.2	18.9	18.3	17.4	15.1	20.3	14.9	19.2	16.6
18.5-24.9	69.9	71.0	74.0	74.2	75.1	75.3	75.2	71.5	75.6	71.8	74.4	72.1	75.1	70.4	70.0
≥25	12.0	14.8	11.4	11.8	8.5	8.1	6.6	9.6	6.1	10.8	10.5	7.6	10.0	10.4	13.5
Parity															
0	40.7	37.1	45.2	39.3	40.2	42.8	43.9	42.9	40.9	38.4	45.5	42.4	42.9	43.3	33.7
1	42.3	39.6	35.1	40.8	40.4	36.9	40.1	37.6	36.6	40.4	37.2	36.6	38.6	34.9	34.6
≥2	17.0	23.3	19.7	20.0	19.5	20.3	16.1	19.6	22.6	21.3	17.4	21.0	18.6	21.7	31.8
Infertility treatment															
No	94.7	97.4	95.9	96.2	96.1	93.9	93.1	88.9	95.7	93.0	92.8	88.9	95.6	94.6	96.0

SD, standard deviation.

[a]Missing values were removed.
