# Supplementary material for: The Japan Environment and Children’s Study (JECS): A Preliminary Report on Selected Characteristics of Approximately 10 000 Pregnant Women Recruited During the First Year of the Study
Source: J Epidemiol. 2015 Jun 5;25(6):452–8. doi: 10.2188/jea.JE20140186 (PMC4444500; doi:10.2188/jea.JE20140186)
Supplement: eTable 3. [file je-25-452-s003.pdf]

eTable 3. Selected infant characteristics according to Regional Centers in the Japan Environment and Children's Study (JECS) as of 2011

Variables[a]	Hokkaido (%)	Miyagi (%)	Fukushima (%)	Chiba (%)	Kanagawa (%)	Koshin (%)	Toyama (%)	Aichi (%)	Kyoto (%)	Osaka (%)	Hyogo (%)	Tottori (%)	Kochi (%)	Fukuoka (%)	South Kyushu /Okinawa (%)
Number of live births	649	1,049	585	538	480	832	706	472	161	1,032	498	310	756	869	698
Singleton births	98.9	97.9	98.6	98.1	99.2	97.8	99.9	94.3	95.0	98.6	96.4	98.7	98.9	97.7	98.9
Gestational age at birth, weeks															
Total, mean (SD)	39.0 (1.7)	39.0 (1.7)	39.3 (1.5)	39.1 (1.5)	39.0 (2.0)	38.9 (1.9)	39.2 (1.6)	38.8 (1.9)	38.7 (2.4)	39.2 (1.8)	38.9 (2.2)	39.3 (1.7)	39.1 (1.8)	39.0 (1.8)	39.0 (1.9)
Preterm births (<37)	6.8	7.5	4.6	6.3	6.9	7.7	4.7	9.8	11.2	5.9	7.2	6.5	6.8	8.8	5.9
Term births (37-41)	93.2	92.5	95.4	93.7	93.1	92.3	94.1	90.3	88.8	93.7	92.4	93.2	93.3	91.1	93.8
Postterm births (≥42)	0.0	0.0	0.0	0.0	0.0	0.0	1.3	0.0	0.0	0.4	0.4	0.3	0.0	0.1	0.3
Sex															
Male	50.5	49.3	54.4	48.1	57.7	50.5	49.4	50.2	54.7	51.7	55.4	43.9	49.7	50.2	52.3
Female	49.3	49.6	45.6	50.9	42.3	49.3	50.6	49.4	44.7	48.4	44.6	56.1	50.3	49.8	47.7
Missing[b]	0.2	1.1	0.0	0.9	0.0	0.2	0.0	0.4	0.6	0.0	0.0	0.0	0.0	0.0	0.0
Type of delivery															
Vaginal	80.3	80.4	83.6	80.3	84.9	82.1	82.9	73.3	72.5	81.9	76.7	81.3	75.7	81.0	80.1
Cesarean	19.7	19.6	16.4	19.7	15.2	17.9	17.1	26.7	27.5	18.1	23.3	18.7	24.3	19.0	19.9
Birth weight, g															
Total, mean (SD)	3,013 (479)	3,024 (445)	3,011 (381)	3,004 (419)	2,971 (441)	2,925 (458)	2,994 (396)	2,949 (473)	2,965 (548)	2,992 (438)	2,969 (504)	3,004 (435)	2,991 (432)	2,953 (443)	3,020 (473)
Singleton births															
Total, mean (SD)	3,024 (467)	3,045 (419)	3,020 (374)	3,016 (413)	2,975 (439)	2,946 (436)	2,994 (397)	2,991 (441)	3,013 (504)	3,002 (430)	2,992 (491)	3,012 (424)	3,002 (416)	2,971 (426)	3,029 (468)
Male, mean (SD)	3,092 (460)	3,069 (418)	3,061 (369)	3,076 (419)	3,029 (420)	3,000 (466)	3,017 (410)	3,019 (421)	3,092 (385)	3,058 (409)	3,027 (487)	3,051 (453)	3,065 (405)	3,008 (424)	3,062 (484)
Female, mean (SD)	2,957 (465)	3,025 (420)	2,972 (376)	2,965 (400)	2,900 (456)	2,887 (394)	2,972 (382)	2,965 (458)	2,912 (611)	2,941 (444)	2,947 (494)	2,981 (399)	2,941 (419)	2,932 (424)	2,994 (449)
Low birth weight (<2,500 g)	9.1	7.6	7.1	7.6	9.8	12.3	8.1	10.6	9.2	8.3	9.4	10.2	8.6	11.0	8.6

SD, standard deviation.

[a]Missing values were removed

[b]Including newborns with ambiguous genitalia
