# Supplementary material for: The Japan Environment and Children’s Study (JECS): A Preliminary Report on Selected Characteristics of Approximately 10 000 Pregnant Women Recruited During the First Year of the Study
Source: J Epidemiol. 2015 Jun 5;25(6):452–8. doi: 10.2188/jea.JE20140186 (PMC4444500; doi:10.2188/jea.JE20140186)
Supplement: Abstract in Japanese. [file je-25-452-s004.pdf]

子どもの健康と環境に関する全国調査（エコチル調査）:
開始 1 年目に参加登録された約 1 万人の妊婦に関する基本属性

道川武紘[1]、新田裕史[1]、中山祥嗣[1]、小野雅司[1]、米元純三[1]、
田村憲治[1]、須田英子[1]、伊藤裕康[1]、竹内文乃[1]、川本俊弘[1,2]
for the Japan Environment and Children’s Study Group

[1] 独立行政法人国立環境研究所　環境健康研究センター　エコチル調査コアセンター
[2] 産業医科大学　医学部　産業衛生学

【背景】
子どもの健康と環境に関する全国調査（エコチル調査）は、2011 年に妊婦の参加登録を開始した環境省が主導する全国規模の出生コホート研究である。この調査の進捗を報告するため、我々は開始 1 年目に集まったデータを用いて、妊婦および出生児の基本属性を集計した。

【方法】
日本全国 15 地域において、産科施設あるいは自治体の母子健康手帳発行窓口にて参加者（妊婦）をリクルートした。妊娠前期に 1 回目の質問票調査、妊娠中期から後期に 2 回目の質問票調査を行い、参加者の基礎情報、身体的精神的な健康状態、生活習慣、職業、生活環境曝露、社会経済状況などの情報を収集した。また、妊娠出産歴、妊娠に関連した病歴、身体計測値、出産記録などの医療情報については、妊娠前期および出産時に実施した診療録転記調査から得た。

【結果】
開始 1 年目の 2011 年に出産した妊婦 9.819 名（平均年齢 31.0 歳）の基本属性をまとめた。生産児は 9,635 名であり、その胎数、在胎期間、性比、出生時体重に関する集計結果は、国の統計と非常に近い結果であった。

【結論】
全ての出産を含んだ出産時データがまとまった時、エコチル調査参加者の国民代表性について最終的な検討を行う予定である。このエコチル調査から得られる成果は、日本の環境が今を生きる子ども達の健康や成長に与える影響について有益な情報を与えるものと考えている。

キーワード：出生コホート、妊婦、子ども、環境化学物質、日本
